# *DYRK1A* pathogenic variants in two patients with syndromic intellectual disability and a review of the literature

**DOI:** 10.1002/mgg3.1544

**Published:** 2020-11-07

**Authors:** Laura E. Meissner, Ellen F. Macnamara, Precilla D'Souza, John Yang, Gilbert Vezina, Undiagnosed Diseases Network, Maria T. Acosta, Margaret Adam, David R. Adams, Pankaj B. Agrawal, Mercedes E. Alejandro, Justin Alvey, Laura Amendola, Ashley Andrews, Euan A. Ashley, Mahshid S. Azamian, Carlos A. Bacino, Guney Bademci, Eva Baker, Ashok Balasubramanyam, Dustin Baldridge, Jim Bale, Michael Bamshad, Deborah Barbouth, Pinar Bayrak‐Toydemir, Anita Beck, Alan H. Beggs, Edward Behrens, Gill Bejerano, Jimmy Bennet, Beverly Berg‐Rood, Jonathan A. Bernstein, Gerard T. Berry, Anna Bican, Stephanie Bivona, Elizabeth Blue, John Bohnsack, Carsten Bonnenmann, Devon Bonner, Lorenzo Botto, Brenna Boyd, Lauren C. Briere, Elly Brokamp, Gabrielle Brown, Elizabeth A. Burke, Lindsay C. Burrage, Manish J. Butte, Peter Byers, William E. Byrd, John Carey, Olveen Carrasquillo, Ta Chen Peter Chang, Sirisak Chanprasert, Hsiao‐Tuan Chao, Gary D. Clark, Terra R. Coakley, Laurel A. Cobban, Joy D. Cogan, Matthew Coggins, F. Sessions Cole, Heather A. Colley, Cynthia M. Cooper, Heidi Cope, William J. Craigen, Andrew B. Crouse, Michael Cunningham, Precilla D'Souza, Hongzheng Dai, Surendra Dasari, Mariska Davids, Jyoti G. Dayal, Matthew Deardorff, Esteban C. Dell'Angelica, Shweta U. Dhar, Katrina Dipple, Daniel Doherty, Naghmeh Dorrani, Emilie D. Douine, David D. Draper, Laura Duncan, Dawn Earl, David J. Eckstein, Lisa T. Emrick, Christine M. Eng, Cecilia Esteves, Tyra Estwick, Marni Falk, Liliana Fernandez, Carlos Ferreira, Elizabeth L. Fieg, Laurie C. Findley, Paul G. Fisher, Brent L. Fogel, Irman Forghani, Laure Fresard, William A. Gahl, Ian Glass, Rena A. Godfrey, Katie Golden‐Grant, Alica M. Goldman, David B. Goldstein, Alana Grajewski, Catherine A. Groden, Andrea L. Gropman, Irma Gutierrez, Sihoun Hahn, Rizwan Hamid, Neil A. Hanchard, Kelly Hassey, Nichole Hayes, Frances High, Anne Hing, Fuki M. Hisama, Ingrid A. Holm, Jason Hom, Martha Horike‐Pyne, Alden Huang, Yong Huang, Rosario Isasi, Fariha Jamal, Gail P. Jarvik, Jeffrey Jarvik, Suman Jayadev, Jean M. Johnston, Lefkothea Karaviti, Emily G. Kelley, Jennifer Kennedy, Dana Kiley, Isaac S. Kohane, Jennefer N. Kohler, Deborah Krakow, Donna M. Krasnewich, Elijah Kravets, Susan Korrick, Mary Koziura, Joel B. Krier, Seema R. Lalani, Byron Lam, Christina Lam, Brendan C. Lanpher, Ian R. Lanza, C Christopher Lau, Kimberly LeBlanc, Brendan H. Lee, Hane Lee, Roy Levitt, Richard A. Lewis, Sharyn A. Lincoln, Pengfei Liu, Xue Zhong Liu, Nicola Longo, Sandra K. Loo, Joseph Loscalzo, Richard L. Maas, Ellen F. Macnamara, Calum A. MacRae, Valerie V. Maduro, Marta M. Majcherska, Bryan Mak, May Christine V. Malicdan, Laura A. Mamounas, Teri A. Manolio, Rong Mao, Kenneth Maravilla, Thomas C. Markello, Ronit Marom, Gabor Marth, Beth A. Martin, Martin G. Martin, Julian A. Martínez‐Agosto, Shruti Marwaha, Jacob McCauley, Allyn McConkie‐Rosell, Colleen E. McCormack, Alexa T. McCray, Elisabeth McGee, Heather Mefford, J. Lawrence Merritt, Matthew Might, Ghayda Mirzaa, Eva Morava, Paolo M. Moretti, Marie Morimoto, John J. Mulvihill, David R. Murdock, Mariko Nakano‐Okuno, Avi Nath, Stan F. Nelson, John H. Newman, Sarah K. Nicholas, Deborah Nickerson, Shirley Nieves‐Rodriguez, Donna Novacic, Devin Oglesbee, James P. Orengo, Laura Pace, Stephen Pak, J. Carl Pallais, Christina G. S. Palmer, Jeanette C. Papp, Neil H. Parker, John A. Phillips, Jennifer E. Posey, Lorraine Potocki, Barbara N. Pusey, Aaron Quinlan, Wendy Raskind, Archana N. Raja, Deepak A. Rao, Genecee Renteria, Chloe M. Reuter, Lynette Rives, Amy K. Robertson, Lance H. Rodan, Jill A. Rosenfeld, Natalie Rosenwasser, Maura Ruzhnikov, Ralph Sacco, Jacinda B. Sampson, Susan L. Samson, Mario Saporta, C. Ron Scott, Judy Schaechter, Timothy Schedl, Kelly Schoch, Daryl A. Scott, Prashant Sharma, Vandana Shashi, Jimann Shin, Rebecca Signer, Catherine H. Sillari, Edwin K. Silverman, Janet S. Sinsheimer, Kathy Sisco, Edward C. Smith, Kevin S. Smith, Emily Solem, Lilianna Solnica‐Krezel, Rebecca C. Spillmann, Joan M. Stoler, Nicholas Stong, Jennifer A. Sullivan, Kathleen Sullivan, Angela Sun, Shirley Sutton, David A. Sweetser, Virginia Sybert, Holly K. Tabor, Cecelia P. Tamburro, Queenie K.‐G. Tan, Mustafa Tekin, Fred Telischi, Willa Thorson, Cynthia J. Tifft, Camilo Toro, Alyssa A. Tran, Brianna M. Tucker, Tiina K. Urv, Adeline Vanderver, Matt Velinder, Dave Viskochil, Tiphanie P. Vogel, Colleen E. Wahl, Stephanie Wallace, Nicole M. Walley, Chris A. Walsh, Melissa Walker, Jennifer Wambach, Jijun Wan, Lee‐kai Wang, Michael F. Wangler, Patricia A. Ward, Daniel Wegner, Mark Wener, Tara Wenger, Katherine Wesseling Perry, Monte Westerfield, Matthew T. Wheeler, Jordan Whitlock, Lynne A. Wolfe, Jeremy D. Woods, Shinya Yamamoto, John Yang, Guoyun Yu, Diane B. Zastrow, Chunli Zhao, Stephan Zuchner, Carlos R. Ferreira, Wadih M. Zein, Cynthia J. Tifft, David R. Adams

**Affiliations:** ^1^ Office of the Clinical Director National Human Genome Research Institute NIH Bethesda MD USA; ^2^ Undiagnosed Diseases Program The Common Fund NIH Bethesda MD USA; ^3^ Division of Diagnostic Imaging and Radiology Children's National Health System Washington DC USA; ^4^ Common Fund Office of the Director National Institutes of Health Bethesda MD USA; ^5^ Medical Genomics and Metabolic Genetics Branch National Human Genome Research Institute National Institutes of Health Bethesda MD USA; ^6^ Ophthalmic Genetics and Visual Function Branch National Eye Institute NIH Bethesda MD USA; ^7^ Present address: Sidney Kimmel Medical College at Thomas Jefferson University Philadelphia PA USA

**Keywords:** autosomal dominant mental retardation 7, Down syndrome, DYRK1A, feeding difficulties, microcephaly

## Abstract

**Background:**

*DYRK1A*‐Related Intellectual Disability Syndrome is a rare autosomal dominant condition characterized by intellectual disability, speech and language delays, microcephaly, facial dysmorphism, and feeding difficulties. Affected individuals represent simplex cases that result from de novo heterozygous pathogenic variants in *DYRK1A* (OMIM 614104), or chromosomal structural rearrangements involving the *DYRK1A* locus. Due to the rarity of *DYRK1A*‐Related Intellectual Disability Syndrome, the spectrum of symptoms associated with this disease has not been completely defined.

**Methods and results:**

We present two unrelated cases of *DYRK1A*‐Related Intellectual Disability Syndrome resulting from variants in *DYRK1A*. Both probands presented to the National Institutes of Health (NIH) with multiple dysmorphic facial features, primary microcephaly, absent or minimal speech, feeding difficulties, and cognitive impairment; features that have been previously reported in individuals with *DYRK1A*. During NIH evaluation, additional features of enlarged cerebral subarachnoid spaces, retinal vascular tortuosity, and bilateral anomalous large optic discs with increased cup‐to‐disc ratio were identified in the first proband and multiple ophthalmologic abnormalities and sensorineural hearing loss were identified in the second proband.

**Conclusion:**

We recommend that the workup of future of patients include a comprehensive eye exam. Early establishment of physical, occupational, and speech therapy may help in the management of ataxia, hypertonia, and speech impairments common in these patients.

## Graphical abstract

Two patients diagnosed with DYRK1A Syndrome in the Undiagnosed Diseases Program with ophthalmologic abnormalities not previously described.

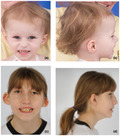

## 1 INTRODUCTION

Autosomal dominant mental retardation 7 (MRD7), or *DYRK1A*‐Related Intellectual Disability Syndrome (OMIM 614104), results from de novo variants in, or structural rearrangements that overlap the *DYRK1A* locus (van Bon et al., 2016). Located within the Down syndrome (DS) critical region on chromosome 21q22.13, *DYRK1A* has been studied extensively in animals to elucidate its role in Down syndrome (Bronicki et al., 2015). *DYRK1A* spans a 150 kDa region and contains 11 coding exons that are alternatively spliced, generating at least five protein isoforms. As a member of the highly conserved dual‐specificity tyrosine‐(Y)‐phosphorylation‐regulated kinase family, *DYRK1A* contains a protein kinase domain, leucine zipper motif, nuclear targeting signal sequence, and a conserved 13‐consecutive‐histidine repeat (Soundararajan et al., 2013). DYRK1A phosphorylates target proteins on serine and threonine residues and undergoes autophosphorylation (van Bon et al., 2016).

DYRK1A interacts with an extensive network of proteins and is implicated in the regulation of apoptosis and proliferation, immunity, cardiovascular function, and neural function (Kay et al., 2016). The neural function of DYRK1A is the most well characterized, and previous work has shown a dosage‐dependent role in neurogenesis, neurodegeneration, neuronal proliferation and differentiation, neuritogenesis, dendritic and spine growth, and synaptic transmission (Dang et al., 2018; Kay et al., 2016; Park & Chung, 2013). Additionally, perturbation of DYRK1A function has been associated with neurodegenerative diseases, such as Alzheimer's disease (AD) and Parkinson's disease (PD) (Wegiel et al., 2011).

DYRK1A has been implicated in contributing to the features of DS and its inhibition by small molecule drugs is of therapeutic interest. The kinase domain of DYRK1A has been the target of specific ATP‐competitive small inhibitors that have been effective in rescuing DS features and improving AD pathology in a murine model (Branca et al., 2017; Kim et al., 2016). However, the de novo mutations in DYRK1A seen in MRD7 produce haploinsufficiency, suggesting a loss‐of‐function mechanism. As a result, the clinical utility of inhibition therapy remains unclear. Here, we describe two unrelated cases of DYRK1A, one with a novel variant and one who's variant has been previously described in association with autism phenotypes (Earl et al., 2017).

## 2 PATIENTS AND METHODS

### 2.1 Ethical compliance

The probands were evaluated at the NIH under the Protocols 15‐HG‐0130, “Clinical and Genetic Evaluation of Patients with Undiagnosed Disorders Through the Undiagnosed Diseases Network,” and 76‐HG‐0238, “Diagnosis and Treatment of Patients with Inborn Errors of Metabolism or Other Genetic Disorders,” both approved by the National Human Genome Research Institute Institutional Review Board. In both cases, extensive phenotyping was performed and biospecimens were collected following written informed consent from both parents.

DNA samples extracted from whole blood were provided for trio exome sequencing from the proband, mother, and father to Baylor Miraca Genetics Laboratories for proband 1 and the NIH Intramural Sequencing Center for proband 2.

### 2.2 Proband 1

The female proband was born to a nonconsanguineous 36‐year‐old G4P3AB1 mother and 38‐year‐old father at 36.5 weeks' gestation via repeat cesarean section with APGAR scores of 8 and 9 at 1 and 5 minutes, respectively. The pregnancy was complicated by Intrauterine Growth Retardation (IUGR) noted at 20 weeks gestation. At birth, the infant was small for gestational age with head circumference of 30.5 cm (6th centile), weight 2.098 kg (5th centile), and length of 42.5 cm (3rd centile). The proband was transferred to the NICU for oral feeding difficulties, poor weight gain, gastroesophageal reflux disease (GERD), and was discharged at day 14 of life.

At 1 month of age, the proband was found to have a heart murmur, and an echocardiogram revealed mild pulmonary artery branch stenosis. Decreased axial tone and global developmental delays were reported at 6 months of age, with gross motor more severely affected than fine motor. She had generalized stiffness and was unable to roll until 9 months of age when physio‐ and occupational therapy were initiated. She sat at 12 months, stood at 15 months, and walked independently at 18 months of age. Babbling was delayed until 20 months of age.

The history is also significant for bilateral hyperopia and alternating esotropia with left eye strabismic amblyopia, for which she was successfully treated with patching then surgery at 15 months of age.

**FIGURE 1:**
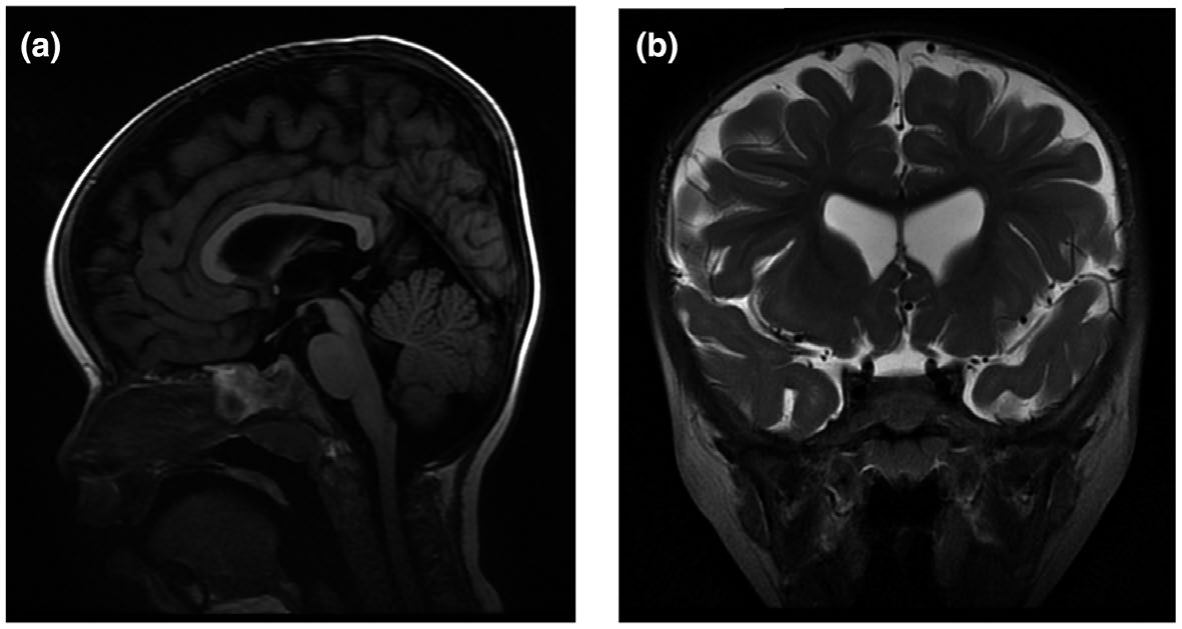
Magnetic resonance imaging of the brain. (a) sagittal and (b) coronal plane showing widened cerebral subarachnoid space, cerebral ventriculomegaly, and generalized mild cerebral cortex volume loss

At 2 years, she experienced a febrile seizure. Magnetic resonance imaging at 2 years 3 months showed widened subarachnoid spaces, ventriculomegaly, and mild cerebral volume loss (Figure 1).

A diagnosis of Russell–Silver syndrome was entertained, though methylation studies were unrevealing. Genetic testing prior to her admission to NIH was nondiagnostic and included Russell–Silver uniparental disomy studies and a proportionate short stature/small for gestational age panel (44 genes). A maternally inherited variant of unknown significance in *KMT2D* was previously identified, though her presentation was not consistent with Kabuki syndrome. An endocrinology evaluation did not show evidence of growth hormone deficiency; however, growth hormone therapy was initiated based on a postnatal standing height of <1% at 2 years 4 months. All other systems including audiology, hematology, and respiratory were reported to be normal.

**FIGURE 2:**
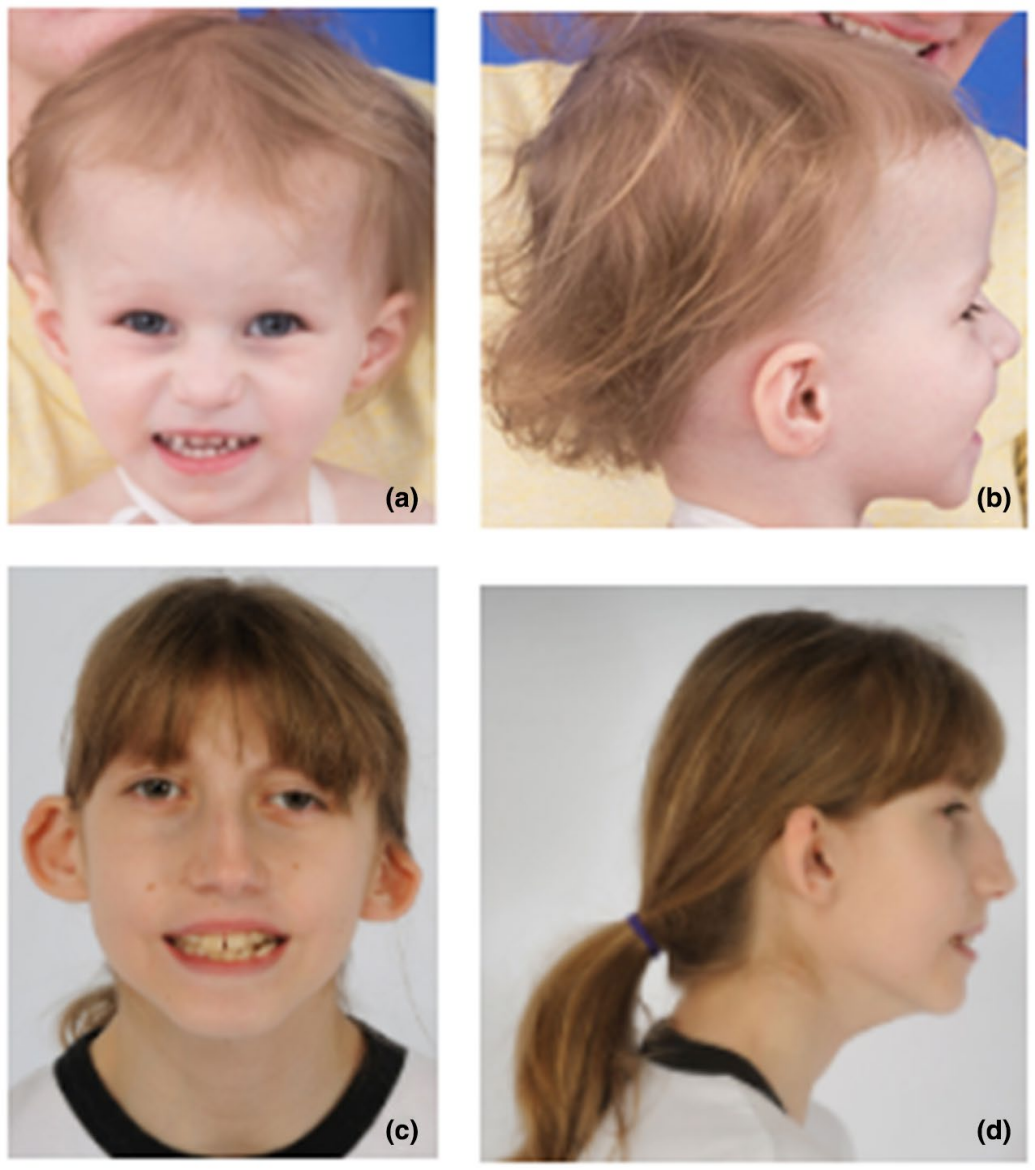
(a) and (b) Proband 1 at 2 years, 7 months. Image displays typical facial gestalt including microtia, deeply set eyes, thin upper lip vermilion, flat philtrum, and triangular face. (c) and (d) Proband 2 at 12 years, 8 months. Image displays narrow facies, low‐set ears with cupped right ear and simple deformed helix on the left, mild hypertelorism, and mid‐face malar hypoplasia

The proband was admitted to the Undiagnosed Diseases Program (UDP) at 2 years 7 months to identify a comprehensive diagnosis. The evaluation revealed dysmorphic facial features including microtia, deep set eyes, thin upper lip vermilion, flat philtrum, triangular face, dental crowding, high, narrow palate, flat occiput, prominent forehead, and midface retrusion (Figure 2). The growth parameters included weight (−4.25 SD), height (*Z* score −3.68 SD), and head circumference (−2 SD), with a body mass index between the 10th and 25th centiles.

An ophthalmological evaluation revealed deep‐set eyes, orthotropia following strabismus surgery, tortuous retinal vessels, bilateral anomalous optic nerves with increased cup‐to‐disc ratio, and mild hyperopic astigmatism. Cardiac evaluation showed mild narrowing of the left pulmonary artery.

Neurological examination revealed mild decreased axial tone and positive scarf sign. Neurodevelopmental evaluation identified delays in motor, cognitive and adaptive skills, and language domains diagnostic of global developmental delay, with the most significant delay being receptive language.

Radiological testing including a skeletal survey, bone age, and abdominal ultrasound were within normal limits.

Laboratory evaluation identified iron deficiency anemia, anemia of chronic disease, and deficiencies of vitamin D and carnitine.

### 2.3 Proband 2

The second female proband was born to a nonconsanguineous 20‐year‐old G1P0 mother and a 24‐year‐old father at 39 weeks gestation by spontaneous vaginal delivery, with APGAR scores of 7 and 9, at 1 and 5 minutes, respectively. No prenatal imaging was performed. At birth, the infant was small for gestational age with a head circumference of 29 cm (−3.8 SD), weight 2.19 kg (−2.8 SD), and length of 43.5 cm (−2.9 SD). The infant had a weak cry and had several cyanotic episodes within the first several hours after birth.

At 2 months of age, an echocardiogram showed a small pulmonary valve and a 7 mm‐atrial septal defect that closed spontaneously. The proband sat at 8 months, crawled at 12 months, and walked at 18 months. She struggled with feeding and remained small for age. An endocrine evaluation at 20 months showed only a slightly high TSH and delayed bone age. Ophthalmology evaluation at 4 years of age diagnosed right superior oblique palsy and left‐sided amblyopia.

Her history is significant for three febrile seizures with normal EEGs. MRI performed at 3 years, 5 months showed a mild prominence of subarachnoid spaces, a small cerebellar vermis, and concern for a small brain stem. Hematologic evaluation at 4 years, 11 months identified immune thrombocytopenia purpura with platelets as low as 61,000 per microliter. Testing for Fanconi anemia was normal.

A diagnosis of CHARGE syndrome was considered, but sequencing and deletion/duplication of *CHD7* was negative. A karyotype, CGH array, subtelomeric FISH, and *SALL4* testing were normal.

The proband was accepted for a comprehensive evaluation by the UDP at 12 years, 8 months. Identified were dysmorphic facial features including right plagiocephaly and a flat occiput, narrow facies, low‐set ears with cupping on the right and deformed helix on the left, jaw asymmetry, dental crowding, ptosis, and mild hypotelorism. The growth parameters included low weight, 27.1 kg (−2.78 SD), a height of 139.8 cm (−2.16 SD), and head circumference of 45.8 cm (−5.5 SD). The body mass index was 13.9 (−2.63 SD).

Ophthalmologic evaluation identified a right superior oblique palsy, bilateral optic nerve head hypoplasia, myopic astigmatism, exotropia, microcornea, and microphthalmia. Audiologic evaluation was suggestive for a mild high‐frequency sensorineural hearing loss. Neurologic examination was significant for truncal hypotonia, hamstring, wrist, and ankle contractures, scoliosis, and global developmental delay. Speech was at a 12‐month‐old level.

A skeletal survey showed microcephaly with overgrowth of the frontal teeth and impaction of multiple teeth. Laboratory evaluation identified elevated blood urea nitrogen and mildly low platelets (157,000 per microliter).

## 3 RESULTS

Trio exome sequencing through Baylor Miraca Genetics Laboratories identified a novel, de novo variant in *DYRK1A*, (NM_001396) in proband 1. The variant, c.201_204delTAAC (p.N68Rfs*2) is absent in gnomAD and is assessed as pathogenic using ACMG criteria.

Trio exome sequencing was performed by NISC and research analysis identified a de novo variant in *DYRK1A*, in proband 2. The variant, c.1401_1402insG (p.I468Dfs*17) is absent in gnomAD and is assessed as pathogenic using ACMG criteria.

## 4 DISCUSSION

The reported incidence of MRD7 is less than 1/1,000,000. It is estimated that in 0.1%–0.5% of individuals with intellectual disability and/or autism, MRD7 is the underlying condition (van Bon et al., 2015). MRD7 is a rare disease with 81 patients previously reported in the literature; 19 of these patients represent structural rearrangements that also impact adjacent genes (Fujita et al., 2010; Matsumoto et al., 1997; Oegema et al., 2010; Valetto et al., 2012). There is no known gender or regional skewing of incidence. Given the rarity of MRD7, there are very few cohorts of patients described in the literature, and no report presents more than 19 patients.

Diagnostic criteria for MRD7 have not been defined, but the disorder should be suspected in individuals with intellectual disability, microcephaly, speech difficulties, feeding difficulties, and the typical facial dysmorphism including microtia, deeply set eyes, thin upper lip vermilion, and flat philtrum. We report two cases of MRD7 in patients with less common features including short stature, pulmonary artery branch stenosis, strabismus, GERD, and cerebral ventriculomegaly. Additionally, retinal vascular tortuosity, bilateral anomalous large optic discs, increased cup‐to‐disc ratio in proband 1, and enlarged subarachnoid spaces in both probands were identified, which have not been previously reported in association with MRD7.

Our cases expand the clinical features associated with MRD7. Increased awareness of MRD7 among health‐care professionals will likely increase the diagnostic rate. Additionally, comprehensive evaluations of patients with this syndrome will further define the disease spectrum. Specifically, these cases demonstrate the value of a full ophthalmologic evaluation for patients suspected of having MRD7. A richer complement of diagnostic criteria for MRD7 may also result in the inclusion of *DYRK1A* in more sequencing panels and completion of the diagnostic odyssey for a greater number of patients.

## CONFLICT OF INTEREST

The authors declare that they have no competing interests.

## AUTHORS' CONTRIBUTIONS

Laura E. Meissner designed, planned, and drafted the manuscript. Ellen F. Macnamara, Precilla D'Souza, Cynthia J. Tifft contributed to the writing and revising of the manuscript for important intellectual content. Precilla D'Souza, Cynthia J. Tifft, David R. Adams, John Yang, Carlos R. Ferreira, Wadih M. Zein were involved in patient evaluation and management. Gilbert Vezina interpreted the MRI findings. Ellen F. Macnamara provided genetic counseling. All authors gave final approval of the version to be published and agree to be accountable for all aspects of the work in ensuring that questions related to the accuracy or integrity of any part of the work are appropriately investigated and resolved.

## Supporting information

Table S1
