# Supplementary material for: DYRK1A pathogenic variants in two patients with syndromic intellectual disability and a review of the literature
Source: Mol Genet Genomic Med. 2020 Nov 7;8(12):e1544. doi: 10.1002/mgg3.1544 (PMC7767569; doi:10.1002/mgg3.1544)
Supplement: Supplementary file 1 — Table S1 [file MGG3-8-e1544-s001.docx]

Supplementary Table 1

Summary of Clinical Features of patients with DYRK1A-associated ID

|  | Proband 1 | Proband 2 | Total |
| --- | --- | --- | --- |
| Intrauterine Growth Retardation | + | Small for gestational age | 19/39  [Møller et al., 2008, Ji et al., 2015, Luco et al., 2016, Murray et al., 2017, Evers et al., 2017, Kim et al., 2017] |
| Primary/Acquired Microcephaly | + | + | 78/81  [Murray et al., 2017, Earl et al., 2017, Evers et al., 2017, Kim et al., 2017] |
| Speech Delay/Absence | + | + | 69/77  [Murray et al., 2017, Earl et al., 2017, Evers et al., 2017] |
| Feeding Difficulties | + |  | 52/55  [Murray et al., 2017, Earl et al., 2017] |
| Typical Facial Gestalt | + | + | 71/72  [Murray et al., 2017, Earl et al., 2017, Evers et al., 2017, Kim et al., 2017] |
| Epilepsy (including Febrile Seizure in Infancy) | + | + | 52/78  [Murray et al., 2017, Earl et al., 2017, Evers et al., 2017] |
| Short Stature | + | + | 14/15  [Ji et al., 2015, Kim et al., 2017] |
| Intellectual disability | + | + | 81/81  [Murray et al., 2017, Earl et al., 2017, Evers et al., 2017, Kim et al., 2017] |
| Stereotypies |  |  | 26/45  [Earl, 2017] |
| Autism Spectrum Disorder |  |  | 22/62  [Earl, 2017, Evers, 2017, Kim, 2017] |
| Anxiety |  |  | 12/44  [Earl et al., 2017] |
| Hyperactivity |  |  | 14/43  [Earl et al., 2017] |
| Hypertonia | + |  | 10/28  [van Bon et al., 2016, Evers et al., 2017] |
| Motor Difficulties including Gait Disturbances | + | Mild dysmetria | 52/53  [Earl et al., 2017] |
| Infection Susceptibility |  |  | 10/24  [Bronicki et al., 2015, Ji et al., 2015] |
| Skeletal Abnormities | + | + | 31/45  [Murray et al., 2017, Earl et al., 2017, Evers et al., 2017] |
| Gastrointestinal Issues | + | + | 14/35  [Møller et al., 2008, Ji et al., 2015, van Bon et al., 2016, Murray et al., 2017, Evers et al., 2017] |
| Endocrinology Issues | + |  | 4/14  [Ji et al., 2015] |
| Abnormal Brain Magnetic Resonance Imaging | + | + | 26/42  [Bronicki et al., 2015, Møller et al., 2008, Ji et al., 2015, Luco et al., 2016, Murray et al., 2017, Evers et al., 2017, Kim et al., 2017] |
| Cardiac Abnormalities | + | + | 7/32  [van Bon et al., 2016, Luco et al., 2016, Murray et al., 2017, Evers et al., 2017] |
| Sleep Disturbances |  |  | 5/9  [van Bon et al., 2016, Luco et al., 2016] |
| Ophthalmologic Abnormalities | + | + | 45/62  [Murray et al., 2017, Earl et al., 2017, Evers et al., 2017] |
